# Age-Related Trends of Caries Experience Among Preschool Children in Shenzhen, China: A Cross-Sectional Study

**DOI:** 10.3390/dj14030149

**Published:** 2026-03-06

**Authors:** Anthony Yihong Cheng, Yuanyuan Liu, Faith Miaomiao Zheng, Ivy Guofang Sun, Jieyi Chen, Chun Hung Chu

**Affiliations:** 1 Faculty of Dentistry, The University of Hong Kong, Hong Kong SAR 999077, China; ayhcheng@connect.hku.hk (A.Y.C.); zhengmm@connect.hku.hk (F.M.Z.); ivysun24@hku.hk (I.G.S.); 2 School of Public Health, Shanxi Medical University, Taiyuan 030001, China; liuyuanyuan1765@sxmu.edu.cn; 3 Guanghua School of Stomatology, Hospital of Stomatology, Sun Yat-sen University, Guangzhou 510055, China

**Keywords:** caries, childhood, preschool, child, prevention, epidemiology, oral health, public health, China

## Abstract

**Objectives**: This study aimed to characterize the age-related trends of caries experience among preschool children in Shenzhen, China. **Methods**: This cross-sectional study recruited 3- to 5-year-old preschool children in Shenzhen via a multistage random sampling method in 2024. Two calibrated examiners conducted oral examinations in kindergartens using disposable dental mirrors with LED illumination and ball-ended Community Periodontal Index probes. Caries experience was recorded using the dmft index, as recommended by the World Health Organization. **Results**: This study invited 4015 children from 27 selected kindergartens, and 3534 children (1886 boys, 53%) completed the survey. The response rate was 88%. The prevalence of caries experience was 31% at age 3, 49% at age 4, and 58% at age 5, representing a 27% higher prevalence in 5-year-olds than in 3-year-olds. The mean dmft scores (±SD) were 1.2 ± 2.5 for 3-year-olds, 2.2 ± 3.2 for 4-year-olds, and 2.8 ± 3.5 for 5-year-olds, indicating a 1.6 affected teeth higher mean dmft in 5-year-olds than in 3-year-olds. The upper central incisors were the most affected teeth (23%) in 3-year-olds and remained the most susceptible across all age groups, while the prevalence of caries in lower molars increased progressively from 7% at age 3 to 24% at age 5. **Conclusions**: Dental caries prevalence and severity among preschool children in Shenzhen increase significantly with age. These findings highlight the need for targeted preventive strategies focusing on high-risk teeth, including the upper central incisors and lower molars, to address the rising burden of early childhood caries in this population.

## 1. Introduction

Dental caries is a chronic disease characterized by the demineralization of tooth hard tissues due to acid produced by cariogenic bacteria [1]. Individuals are susceptible throughout life, yet young children are particularly vulnerable because they have limited manual dexterity and cannot remove plaque effectively without caregiver support [2]. Frequent consumption of sugary snacks or beverages further increases caries risk [3]. Early childhood caries is multifactorial, commonly driven by the interaction of poor oral hygiene, cariogenic dietary patterns, and changes in the oral microbial flora [4]. Following eruption of primary teeth, non-shedding tooth surfaces enable plaque biofilm establishment and maturation [5]. With dietary diversification and increased exposure to fermentable carbohydrates, biofilms may shift toward a more acidogenic and acid-tolerant composition, increasing susceptibility to caries in preschool children [6].

The global epidemiological landscape of dental caries among preschool children is alarming, with a pooled prevalence rate of 48%, ranging from 30% to 82% [7]. In mainland China, the situation is equally concerning, with the latest National Oral Health Survey (2015–2016) reporting dental caries experience rates of 51% among 3-year-olds, 64% among 4-year-olds, and 72% among 5-year-olds [8]. The consequences of severe dental caries are significant and multifaceted, encompassing pain, infection, premature loss of primary teeth, malocclusion, nutritional deficits, and psycho-social issues that collectively impact children’s overall well-being and quality of life [9,10]. Given the high prevalence and adverse sequelae of dental caries among preschool children, an updated epidemiological survey is essential to elucidate the current status and developmental trends of this condition.

Shenzhen is a coastal city in southern China. It has garnered global attention for its remarkable economic expansion and technological breakthroughs. Established as China’s first Special Economic Zone in 1980, Shenzhen has undergone a dramatic transformation from a modest fishing village to a globally influential metropolis [11]. It is referred to the China’s Silicon Valley because it is a center for technological innovation, hosting leading high-tech enterprises such as Tencent, Huawei, DJI and ZTE [12]. Additionally, Shenzhen Stock Exchange plays a crucial role in driving China’s economic prosperity [13]. This development has attracted sustained internal migration. As a result, Shenzhen’s population is large, relatively young, and highly mobile. Many families are recent arrivals, and the city includes both long-term residents and migrant households [14]. This creates marked socioeconomic and cultural diversity across communities and kindergartens. As of 2024, Shenzhen is home to approximately 590,000 children enrolled in over 1900 kindergartens [15]. The rapid population growth has also exerted considerable pressure on the healthcare system, particularly in addressing children’s oral health. Understanding the current situation is essential for developing precisely targeted interventions that cater to the distinct needs of each age group and ultimately enhance children’s oral health outcomes [16]. This study aimed to characterize the age-related trends of caries experience among preschool children in Shenzhen, China.

## 2. Materials and Methods

This cross-sectional study was conducted from March to May 2024 in Shenzhen. Ethical approval was obtained from the University of Hong Kong/Hospital Authority Hong Kong West Cluster (IRB: UW 23-064) and the Medical Ethics Committee of Shenzhen Center for Disease Control and Prevention (IRB: QS2023020059). The study’s reporting framework was structured to conform to the guidelines outlined in the STROBE (Strengthening the Reporting of Observational Studies in Epidemiology) statement (Supplementary Table S1) [17].

### 2.1. Clinical Examination

Two trained and calibrated dentists conducted the dental examinations. They received training to diagnose dental caries at the cavitation level from an experienced dental epidemiologist to ensure high inter- and intra-examiner reliability, with a kappa value exceeding 0.9. Written informed consent was obtained from the parents or legal guardians of all participating children prior to examination. Dental examinations were performed in kindergartens using disposable dental mirrors with LED illumination and ball-ended Community Periodontal Index probes, with children positioned in the supine position. Caries experience was recorded using the dmft (decayed, missing, and filled teeth) index, as recommended by the World Health Organization (WHO) [18]. To monitor diagnostic reliability, approximately 10% of the children were randomly selected for re-examination on each revisit by a research assistant without prior notification to the examiners. Individual oral health reports were provided to each child’s parent, and parents were informed that they could seek further dental treatment at their own expense if needed.

### 2.2. Sampling Method

This study used a multistage cluster sampling method to recruit children. In the two main districts of Shenzhen, kindergartens include both public and private institutions, with an approximate ratio of 1:1. Both public and private kindergartens were sequentially numbered and selected through simple random sampling. All children in grades K1 to K3 from the selected kindergartens were then invited to participate in the survey. Children whose parents provided consent were enrolled in the study. Children attending special needs education or hospitalized were not included in the examination.

### 2.3. Sample Size Calculation

The sample size for this study was calculated using the following formula, where Z represents the Z-score corresponding to the desired confidence level, P denotes the estimated prevalence, and d signifies the acceptable margin of error (precision) [19]: n = Z^2^ × P × (1 − P)/d^2^.

Based on the Fourth National Oral Health Survey, the estimated prevalence of dental caries was 50%. A confidence level of 95% was chosen, with a confidence interval (C.I.) of 45–55%, and a margin of error set at 0.05. Additionally, an anticipated response rate of 80% was incorporated into the calculations. Using these parameters, a sample size of 480 children was required for each grade. Since the Chinese preschool education system typically includes three grades (K1, K2, and K3), the total number of children to be invited was 1440. Considering that the study involves two main districts in Shenzhen, the overall number of children to be invited was 2880. We invited additional children to ensure the final number examined met or exceeded the minimum target in each grade and in each district, and improved the precision and stability of age-related estimates.

### 2.4. Data Analysis

Data entry was performed by the examiners using Microsoft Office Excel 2019 on personal computers. To ensure accuracy, the data were double-checked and cross-verified by the examiners to prevent errors. Statistical analysis was conducted using SPSS version 27.0 for Windows (IBM Inc., Chicago, IL, USA). Descriptive statistics, including frequencies and percentages, were reported. Intra-examiner reliability was assessed using Kappa statistics. The Chi-square test was used to evaluate group differences in categorical variables. The Mann–Whitney *U*-test and Kruskal–Wallis test were used to evaluate the distribution of dmft score among different groups because the dmft score showed a non-normal distribution. A two-sided *p* < 0.05 was considered statistically significant.

## 3. Results

A total of 4015 children from 27 kindergartens were invited to participate, and 3534 preschool children completed the survey, resulting in a response rate of 88%. Among the participants, 1886 were boys (53%) and 1648 were girls (47%). The children’s ages ranged from 3 to 5 years, with a mean age of 4.2 years. Their caries prevalence was 49%. These 3534 children had a total of 7430 teeth with decayed surfaces, 19 teeth missing due to caries, and 402 teeth filled. Therefore, most of their dental caries cases were left untreated (95%). The mean dmft score (±SD) was 2.22 ± 3.26, and the mean dt score (±SD) was 2.10 ± 3.15 (Table 1). Caries prevalence was higher in boys than in girls (*p* = 0.013). The mean dmft scores for boys and girls were 2.33 ± 3.33 and 2.10 ± 3.17, respectively, and this difference was statistically significant (*p* = 0.042).

**Figure 1:**
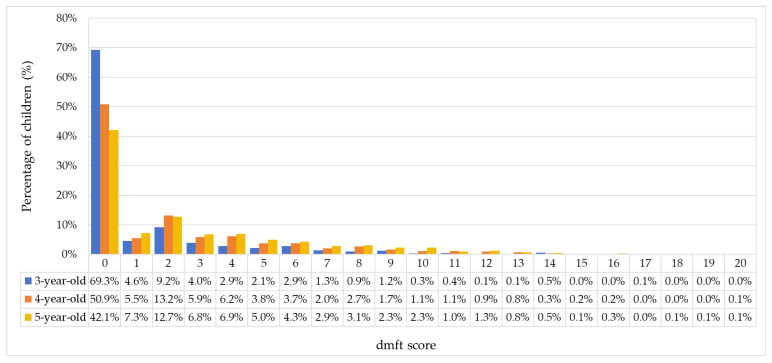
Decayed, missing and filled teeth (dmft) score of Shenzhen children.

The distribution of dmft scores was right-skewed, as shown in Figure 1. A significant proportion of children had a dmft score of zero, indicating no dental caries. Among children with caries experience, the mode dmft score was two. The Significant Caries (SiC) index was 5.80 ± 3.37, calculated as the mean dmft of the one-third of children with the highest dmft scores.

**Figure 2:**
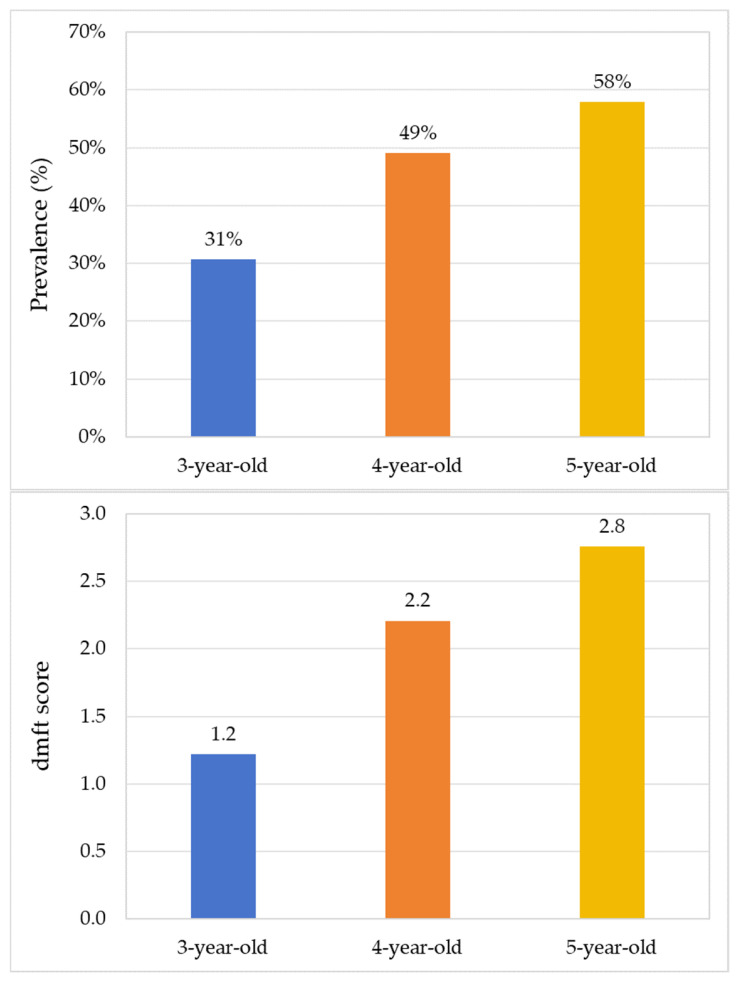
Caries prevalence (upper) and caries experience in dmft (lower) of Shenzhen children.

The prevalence of dental caries increased across age groups: 30.7% in 3-year-olds, 49.1% in 4-year-olds, and 57.9% in 5-year-olds (*p* < 0.001; Figure 2). The mean dmft scores (±SD) also increased across age groups, from 1.22 ± 2.47 in 3-year-olds to 2.76 ± 3.52 in 5-year-olds, with an intermediate value of 2.21 ± 3.21 in 4-year-olds (*p* < 0.001; Figure 2). Given the skewed distribution of dmft, the median (IQR) dmft values were 0 (2) for 3-year-olds, 0 (3) for 4-year-olds, and 2 (4) for 5-year-olds. The SiC scores (±SD) were 3.65 ± 3.07 for 3-year-olds, 5.84 ± 3.18 for 4-year-olds, and 6.88 ± 3.14 for 5-year-olds, indicating a higher caries burden among children with high caries experience in older age groups.

**Figure 3:**
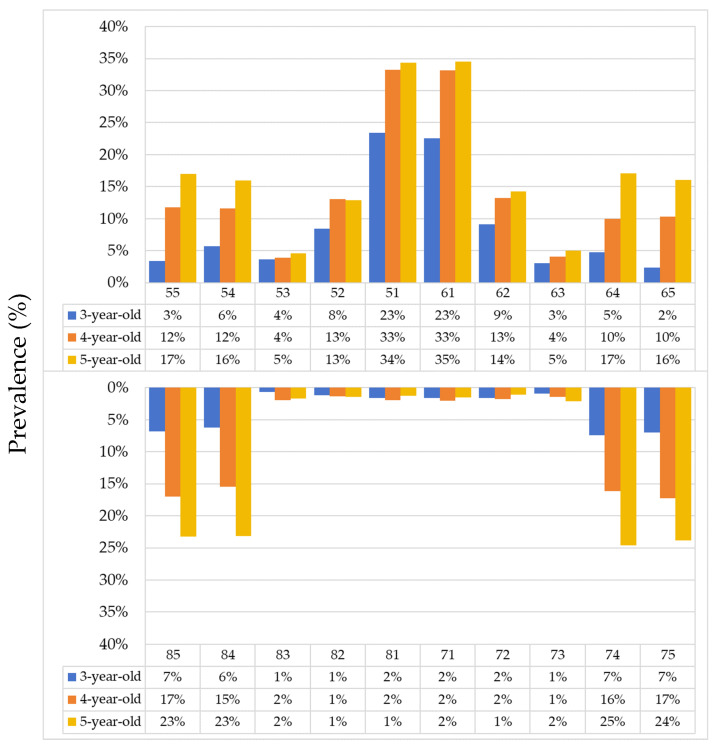
Age-related distribution of carious teeth of Shenzhen children by tooth position.

Among 3-year-olds, the upper central incisors were the most frequently affected teeth, with a prevalence of 23.0%. This pattern remained consistent across all age groups (Figure 3). The prevalence of caries in upper central incisors was higher in older age groups, increasing from 23% in 3-year-olds to 34% in 5-year-olds, indicating an age-related gradient in susceptibility. Lower incisors and canines were less affected, with a prevalence of 1.3% at age 3, and remained below 10% across all age groups. Figure 3 shows the age-related distribution of carious teeth of Shenzhen children by tooth position.

## 4. Discussion

This study reveals a significant uneven distribution of dental caries among preschool children in Shenzhen. Over half of children aged 3 to 4 years and approximately 40% of 5-year-olds remained caries-free, indicating that a substantial portion of this population has good oral health. However, the significant caries index highlights an inequality in the distribution of dental caries. A small subset of children accounted for a disproportionate number of decayed teeth. This phenomenon emphasizes that high-risk groups bear a considerably greater burden of dental caries [20]. Although socioeconomic status and access to dental care were not assessed in this study, these factors have been reported as important contributors to caries inequalities in previous research [21]. In this population, over 90% of teeth affected by caries were left untreated, underscoring the urgent need for improved access to primary dental care services. Addressing this gap may significantly reduce the severity of dental caries and improve oral health outcomes among vulnerable preschool children [22].

Currently, the availability of dental care in Shenzhen remains limited [23]. In 2024, there were 9635 registered dentists in the city, with a dentist-to-population ratio of about 0.18 dentists per 1000 residents [24]. Despite Shenzhen’s rapid economic growth over recent decades, its healthcare infrastructure still faces considerable challenges. Furthermore, a significant proportion of the population—up to 77%—encounters barriers to accessing primary healthcare services [16]. These barriers may include geographic distance, financial constraints, lack of awareness, or insufficient dental clinics, particularly in underserved districts. Although Shenzhen has experienced remarkable economic development, its healthcare system has yet to fully meet the oral health needs of its population. Identifying high-risk children and implementing targeted, accessible oral health interventions are essential steps toward improving the overall oral health status of preschool children and addressing social inequalities.

The observed age-related trends in this study align with global patterns of dental caries development. The prevalence of dental caries among Shenzhen preschool children was higher in older age groups, increasing from 31% in 3-year-olds to 58% in 5-year-olds, indicating a clear age gradient in caries burden. Similar trends have been reported in various regions worldwide, including Northeast Italy [25] and Singapore [26]. Although this cross-sectional study cannot assess incidence or within-child progression, the higher prevalence in older children is consistent with the cumulative nature of caries experience and longer exposure to potential risk factors—such as sugary foods, poor oral hygiene, and frequent consumption of milk bottles—elevates their risk for developing caries [4]. Interestingly, despite geographical proximity and shared socioeconomic characteristics, Hong Kong exhibits notably lower caries prevalence among children [27]. This disparity is largely attributed to public health measures, such as water fluoridation at 0.5 ppm and widespread outreach dental programs utilizing silver diamine fluoride for caries control [28]. These strategies effectively reduce caries incidence and severity [29].

In contrast, Shenzhen’s lower prevalence compared to many other Chinese regions indicates some progress but also highlights ongoing challenges. For example, in Liaoning Province (Northeastern China), the caries prevalence rates are 62% for 3-year-olds and 87% for 5-year-olds [30] and in Zhejiang Province (Eastern China), rates are 60% and 78%, respectively [31]. These disparities reveal significant regional variations in oral health status across China, with many areas experiencing much higher rates than Shenzhen [32]. Given Shenzhen’s relatively better situation, it is reasonable to infer that other less-developed regions may face more severe challenges, emphasizing the need for nationwide targeted interventions to improve oral health outcomes.

Feeding practices and early oral hygiene behaviors are critical determinants of caries risk. Systematic reviews have shown that children breastfed for more than 12 months have approximately twice the risk of developing dental caries compared to those breastfed for shorter durations [33]. Additionally, bottle-feeding has been associated with higher caries prevalence than breastfeeding [34]. These findings underscore the importance of parental education on appropriate feeding behaviors and oral hygiene habits from an early age. Proper guidance can help mitigate early risk factors, such as prolonged exposure to sugary liquids in bottles, which contribute to the initiation of caries, especially on the upper primary incisors—often the first teeth affected. As children grow, the focus of caries susceptibility shifts toward molars, which are more challenging to clean due to their complex anatomy and posterior location.

The FDI World Dental Federation recommends that parents assist with tooth brushing until children develop adequate dexterity and skills to brush effectively independently [35]. Moreover, dental flossing can be a potential approach to reduce plaque accumulation on proximal tooth surfaces, particularly between molars, and may help prevent proximal caries in the primary dentition [36]. However, many parents fail to assist their children with daily flossing, leaving a significant gap in preventive care. Therefore, community-based oral health education programs targeting parents and caregivers are vital to improve daily oral hygiene practices and reduce caries risk.

Proactive prevention should prioritize risk-based, feasible measures that maintain mineral balance and limit cariogenic biofilm in preschool children. Key components include caregiver-supervised twice-daily toothbrushing with fluoridated toothpaste, reduction in the frequency of free-sugar intake (especially sugary drinks and nocturnal bottle-feeding), and early identification of initial lesions during routine kindergarten/community screening. Where appropriate, adjunct remineralization strategies—such as biomimetic hydroxyapatite—may be considered to support enamel repair [37], and selected probiotics may help modulate plaque dysbiosis as a complement to mechanical and chemical plaque control [38].

The strengths of this study include its large sample size and high response rate, which enhance the reliability and generalizability of the findings. The multistage random sampling approach improved representativeness of preschool children in the selected districts and helped minimize selection bias [39]. This methodological rigor provides more accurate estimates of caries prevalence and severity across different age groups. Additionally, the cross-sectional design allowed us to describe age-related trends in dental caries prevalence at a specific point in time, which is useful for identifying high-burden groups and informing public health strategies [40,41]. However, cross-sectional studies cannot establish causality or temporal relationships—such as the development of new cavities without intervention—they are valuable for providing baseline data and understanding the current landscape of oral health among preschool children. Another limitation is that participants were drawn from selected districts; therefore, the findings may not fully represent the entire city, particularly areas with different socioeconomic and population characteristics, and should be generalized with caution. Nonetheless, the data obtained serve as an essential foundation for future research and policy formulation [42].

The findings highlight the urgent need for comprehensive, targeted strategies to reduce the burden of dental caries among preschool children in Shenzhen and across China, particularly among children with high caries experience. Improving access to timely preventive and treatment services is critical to address the substantial unmet treatment needs observed in this study. Public health initiatives should incorporate age-specific prevention programs, parental education on feeding and oral hygiene practices, and community outreach to promote early intervention. Strengthening fluoride use and expanding kindergarten- or community-based oral health services may help reduce caries severity and improve oral health outcomes. Ultimately, coordinated efforts involving policymakers, healthcare providers, educators, and families are necessary to ensure that all children could grow up with healthy, decay-free teeth.

## 5. Conclusions

In Shenzhen, preschool children demonstrated a significant age-related increase in both the prevalence and severity of dental caries. Strengthening comprehensive community-based oral health education and implementing proactive, risk-based prevention, alongside improving access to preventive services and timely treatment, are important to reduce the caries burden and address the substantial unmet treatment needs identified in this study.

## Figures and Tables

**Table 1:** Caries experience (dmft) of 3- to 5-year-old Shenzhen children by age and sex.

Independent Variables (N)	Caries-Free n (%)	Have Dental Caries n (%)	*p*-Value	Mean dt (±SD)	Mean mt (±SD)	Mean ft (±SD)	Mean dmft (±SD)	*p*-Value
All children (3534)	1810 (51.2%)	1724 (48.8%)		2.1 (±3.1)	0.01 (±0.1)	0.1 (±0.6)	2.2 (±3.3)	
Age			<0.001 ^a^					<0.001 ^b^
3-year-old (759)	526 (69.3%)	233 (30.7%)		1.2 (±2.5)	0.0 (±0.1)	0.0 (±0.1)	1.2 (±2.5)	
4-year-old (1313)	668 (50.9%)	645 (49.1%)		2.1 (±3.1)	0.0 (±0.0)	0.1 (±0.5)	2.2 (±3.2)	
5-year-old (1462)	616 (42.1%)	846 (57.9%)		2.5 (±3.4)	0.01 (±0.1)	0.2 (±0.8)	2.8 (±3.5)	
Sex			0.013 ^a^					0.042 ^c^
Female (1648)	881 (53.5%)	767 (46.5%)		2.0 (±3.1)	0.0 (±0.1)	0.1 (±0.6)	2.1 (±3.2)	
Male (1886)	929 (49.3%)	957 (50.7%)		2.2 (±3.2)	0.01 (±0.1)	0.1 (±0.7)	2.3 (±3.3)	

^a^ Chi-square test; ^b^ Kruskal–Wallis test; ^c^ Mann–Whitney *U*-test.

## Data Availability

The results of the dental examination of each participating child will be shared with the parents or legal guardians via oral health reports. The team will share the results of the study with academia via publications and presentations. The datasets generated in this study will be available from the primary investigator on a legitimate request.

## Supplementary Materials

The following supporting information can be downloaded at: https://www.mdpi.com/article/10.3390/dj14030149/s1, Table S1: STROBE Statement—Checklist of items that should be included in reports of cross-sectional studies.

## Abbreviations

The following abbreviations are used in this manuscript:

dmft	decayed, missing, and filled primary teeth
dt	decayed primary teeth
mt	missing primary teeth
ft	filled primary teeth
WHO	World Health Organization
LED	Light-Emitting Diode
STROBE	STrengthening the Reporting of OBservational studies in Epidemiology
IQR	Interquartile Range
IRB	Institutional Review Board
